# Protein unfolding mechanisms and their effects on folding experiments

**DOI:** 10.12688/f1000research.12070.1

**Published:** 2017-09-22

**Authors:** Lisa J Lapidus

**Affiliations:** 1 Department of Physics and Astronomy, Michigan State University, East Lansing, USA

**Keywords:** Protein, Unfolding mechanisms, kinetics

## Abstract

In this review, I discuss the various methods researchers use to unfold proteins in the lab in order to understand protein folding both *in vitro *and *in vivo*. The four main techniques, chemical-, heat-, pressure- and force-denaturation, produce distinctly different unfolded conformational ensembles. Recent measurements have revealed different folding kinetics from different unfolding mechanisms. Thus, comparing these distinct unfolded ensembles sheds light on the underlying free energy landscape of folding.

## Introduction

Ever since Anfinsen discovered that a protein can be reversibly folded and unfolded outside of a cell ^1^, researchers have been investigating the folding process *in vitro*, confident in the knowledge that they were trying to understand a physical process of how the polypeptide chain finds a lowest free energy state. The free energy of the folded state comprises a balance of enthalpy and entropy of the protein and the surrounding solvent. The success of structural biology methods over the past few decades has focused attention on the beautiful structures of the folded states that have been determined for many sequences. In contrast, the unfolded “state”, really an ensemble of disordered conformations, was generally regarded to be highly generic, completely random, and rapidly rearranging ^2, 3^. Recent advances in nuclear magnetic resonance (NMR) and optical methods have started to change this view and detail how different sequences and different solvent conditions can alter the ensemble ^4– 9^.

Since the unfolded state was treated generically, it seemed not to matter how one obtained it, especially if the goal was to leave it as quickly as possible, as is done in kinetic folding experiments. This review will discuss the various methods that are commonly used to unfold proteins and how similar are the resulting ensembles. I will conclude with a discussion of whether the choice of unfolding method then affects the subsequent folding process.

## Chemical denaturants

It remains true that chemical denaturants, guanidine hydrochloride and urea, are the most generally applicable methods of completely unfolding proteins. Both of these molecules have a low molecular weight and are extremely soluble, such that 6–8 molar concentrations will denature virtually any protein. Computational studies of polypeptides interacting with these molecules have revealed some aspects of their denaturing power ^10^, although far more is understood about urea. The molecular dynamics of an unfolded protein indicate that urea readily forms hydrogen bonds with the peptide backbone, disrupts native contacts, and makes extended conformations favorable ^11^. Simulations comparing urea and guanidine on the same protein find that guanidine does not make many hydrogen bonds but does disrupt hydrophobic interactions within the native state, particularly between aromatic side chains ^12^.

A number of studies of various proteins in high denaturant have shown that these chains are acting as self-avoiding random polymers ^13, 14^. Measurement of intramolecular contact of unstructured peptides in water with guanidine and water with urea showed that a wormlike chain with excluded volume is a better model than a freely jointed chain, but the persistence length (4–6 Å) and excluded diameter (4 Å) are sufficiently small that a freely jointed chain is a good model for proteins of any reasonable length ^15, 16^. The intramolecular diffusion coefficients measured by these same experiments reveal values in the 10 ^-6^ cm ^2^/s range for all sequences in high denaturant, about the same as the translational diffusion coefficient for objects of this size ^17– 20^. Thus, the view of unfolded proteins as completely random, freely diffusing polymers appears to be justified in high denaturant.

The difficulty with using denaturant as an unfolding mechanism is the technical challenges with rapidly diluting it to prompt refolding in a kinetic experiment. The dilution or mixing time is a period in which the solution conditions are not in equilibrium and folding kinetics are not in response to a known set of conditions. Conventional stopped-flow mixers have “dead times”, the time during which measurement is not possible, of 1–5 ms that are determined by the turbulence induced in the mixing process, yet folding may still be occurring. Smaller turbulent mixers have pushed this dead time down to as low as 30 μs ^21, 22^. Laminar flow mixers developed in my lab have eliminated turbulence and mix as fast as 2–4 μs ^23– 26^.

## Temperature

It has long been known that proteins generally unfold at temperatures higher than the basal temperature of the organism in which it evolved. Therefore, the melting temperatures of proteins from thermophilic organisms are typically higher than their homologs from mesophilic organisms ^27– 29^. It has been predicted that cold denaturation, protein unfolding that occurs as the temperature is lowered ^30– 36^, is a feature of protein stability, but, practically, the lower melting temperature is rarely observable above 0°C, so water will freeze before the protein will unfold.

The appeal of using heat as a denaturant is that it is completely understood how it affects the protein on the atomic level. It is also the natural denaturant for computational studies, since heat is already accounted for in molecular dynamics simulations. In terms of the relative contributions to the change in the Gibbs free energy between the folded state and unfolded state, *ΔG*= *ΔH-TΔS*, as the temperature increases, *ΔG* will decrease until eventually it becomes less than zero and the unfolded state has a lower free energy than the folded state. However, the absolute free energy of the unfolded state need not remain constant with increasing temperature. In particular, the hydrophobic effect should get stronger with temperature ^37, 38^. Therefore, a protein that is unfolded at an elevated temperature may still have strong intramolecular interactions within the unfolded state that make the unfolded conformations less than completely random.

Temperature is also a natural control variable in experiments. About 20 years ago, the development of laser temperature jump (T-jump) techniques allowed the first observations of protein folding on the ns–μs time scales by adding an IR pulse of light to a protein solution to rapidly raise the temperature ~10°C in ~10 ns ^39– 41^. However, temperature may be only increased because the laser pulse adds heat. Reducing the temperature generally requires a much slower diffusive process that takes milliseconds to equilibrate. Therefore, the kinetic process that is observed is usually dominated by the unfolding process. To extract direct folding rates, researchers have usually used a two-state model of the folding process in which the relative population at each temperature is known from equilibrium measurements ^42– 45^. However, it is possible that a two-state model is not a good approximation of the folding free energy landscape ^46^. Furthermore, given that laser T-jump is limited to increases of ~10°C and most proteins unfold at temperatures well above physiological, neither the start nor the end of the experiment is typically under strong folding conditions (i.e. 37°C).

## Pressure

While few organisms undergo significant changes in pressure over their lifetime, pressure is an attractive unfolding mechanism because, like temperature, it is completely understood physically and can be simulated computationally. Under very high pressures (1–3 kbar or 100–300 MPa), voids within a protein’s folded structure become unstable, causing the protein to unfold ^47^. The contribution to the change in free energy due to pressure is given as *pΔV*. The change in partial molar volume upon unfolding, *ΔV*, is typically negative, making the free energy of the unfolded state lower than the folded state. The kinetics of unfolding are typically extremely slow, orders of magnitude slower than unfolding with denaturant or temperature ^48, 49^. This allows the use of careful NMR measurements to map which residues change structure first and can sometimes find complex folding dynamics. Often intermediates can be detected as well as complete unfolding ^50^. As with T-jump, it is generally difficult to rapidly depressurize to induce refolding. One technique has been demonstrated recently by Dumont *et al*., in which the pressure cell is irreversibly broken for each measurement but achieves depressurization within 1 μs ^51, 52^.

## Force

About 20 years ago, several groups demonstrated that individual molecules could be unfolded using an atomic force microscope or optical tweezers ^53– 55^. In early measurements, proteins were immobilized on a surface and were often constructed of multiple identical domains in tandem in order to confirm the signal was real ^56, 57^. Significant improvements over the past two decades have eliminated the need for a surface (though proteins are now attached to micron-sized beads) and made detection of single-domain unfolding routine, typically using laser tweezers to trap the bead ^58, 59^. At the state of the art, these instruments can detect forces of as low as a few pN of extensions as small as ~4 Å on time scales as short as 10 μs (these limits are convolved) ^60– 62^. Like temperature and pressure, this unfolding mechanism is well understood physically, though the time-range over which these experiments are typically performed (10–100 nm/s stretching speeds) is somewhat longer than typical simulation times. The appeal of these types of measurements is two-fold: 1) they are naturally single-molecule measurements, allowing the researcher to explore heterogeneity in folding pathways ^62– 65^, and 2) the unfolded conformation eventually reached is very well defined, a completely extended polymer ^65^. However, like many single-molecule measurements, they are typically performed at forces near the folding midpoint to allow the observation of many folding and unfolding events. If folding is induced at a low force, the instrument typically has no resolution to observe the event.

## Discussion

As the descriptions above have made clear, each of these unfolding mechanisms produces an ensemble of conformations that is distinct from the others. A logical question is, should this matter? While this question does not yet have a complete answer, there are tantalizing indications that it does. For example, Lin *et al*. measured folding/unfolding kinetics after T-jump to the same final temperature from different initial temperatures ^66^. For a fast two-state folder, tryptophan zipper, the kinetics were the same regardless of the starting condition, but for a protein without a significant free energy barrier between folding and unfolding, BBL, the kinetics were distinguishable. My lab, in collaboration with Bill Eaton’s lab at the NIH, demonstrated that the folding rates of the villin headpiece subdomain (HP35), one of the fastest known folders, are 5-fold slower for folding after dilution of denaturant compared to laser T-jump ^67^. We explained this discrepancy by the Thruway Search Model first described by Ghosh and Dill ^68^, in which the number of paths to the native state is higher and the intrachain energy of the unfolded state is lower for the chemical-denatured ensemble compared to the heat-denatured ensemble. In a study of another fast-folding protein, lambda repressor, T-jump folding rates are about 2–3-fold higher than rapid mixing folding rates ^69^. In a different mutant of lambda, T-jump and P-jump (dropping the pressure from 1 kbar to 1 bar) yield different kinetics that were attributed to a misfolded packing of one helix accessible only when exiting out of the high-pressure unfolded state ^52^. These discrepancies are not a universal phenomenon: T-jump and P-jump of the Fip35 fragment of a WW domain yielded very similar kinetics, as does T-jump and rapid dilution of denaturant of a different WW domain, Pin1 ^70, 71^.

**Figure 1.**
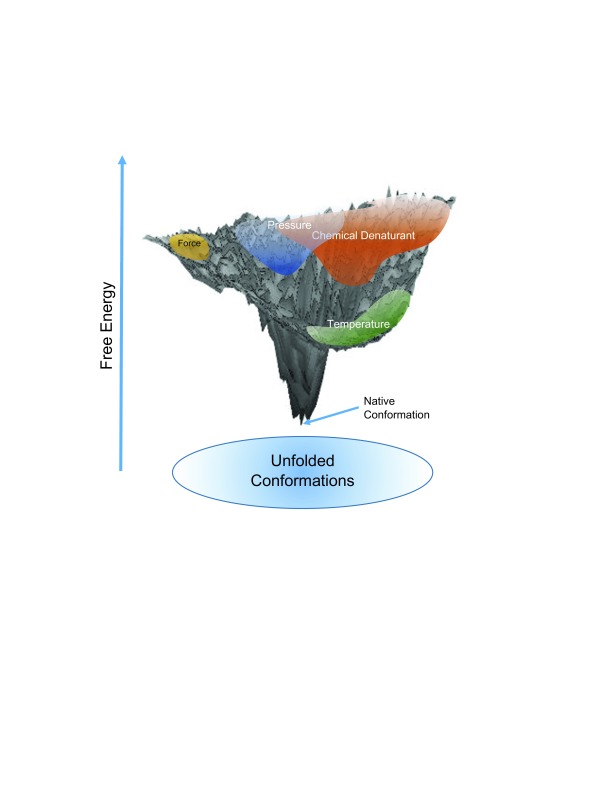
Schematic of the free energy landscape of folding of a protein. Unfolded ensembles by different techniques occupy distinct regions on the unfolded landscape.

A comprehensive view of these comparisons between unfolded ensembles would suggest the complete ensemble of an unfolded protein is very broad and these various unfolding mechanisms are populating only a subset of the ensemble. Putting this in an energy landscape picture, as shown schematically in Figure 1, the top of the protein funnel is very wide and not entirely accessible by all methods. This also means that the approach to the native state from different sides of the funnel may yield different predominant pathways and folding kinetics.

Thus, from a physical perspective, the protein folding problem looks very different for any particular protein depending on how (or from where) you start the process. But from a biological perspective, should one care? The study of protein folding *in vivo* is still in its infancy, so we have very little idea what the unfolded ensemble looks like in a cell. Various studies have shown that thermal stability can shift up or down in crowded *in vivo* conditions compared to dilute *in vitro* conditions, depending on the sequence ^72– 74^. Danielsson and colleagues have speculated that the devil is in the details: exactly how strongly the unfolded ensemble prefers to interact with nearby proteins in a cell will determine how stability will shift ^75^. Furthermore, it is easy to imagine that the conformational ensemble of a nascent chain emerging from the ribosome looks distinct from the ensemble achieved after unfolding within a chaperone such as GroEL/GroES. Therefore, we may expect initial folding and refolding of the same protein to use different pathways. From the perspective of biology, we should very much care how a protein is unfolded.

In conclusion, until we have a completely predictive model of protein folding, in which all folding pathways and the final folded structure are predicted by primary sequence, we have to make educated guesses of what unfolded conformations are accessible under certain conditions for a particular protein. It certainly seems sensible to continue to investigate the entire unfolded landscape under a variety of unfolding mechanisms to fully understand how protein folding depends on where you start.

## Abbreviations

NMR, nuclear magnetic resonance; T-jump, temperature jump.
